# Supplementary material for: Haploidentical versus Double-Cord Blood Stem Cells as a Second Transplantation for Relapsed Acute Myeloid Leukemia
Source: Cancers (Basel). 2023 Jan 10;15(2):454. doi: 10.3390/cancers15020454 (PMC9856318; doi:10.3390/cancers15020454)
Supplement: Supplementary file 1 [file cancers-15-00454-s001.zip › cancers-2086158-SupplementaryMaterials_revised_final.pdf]

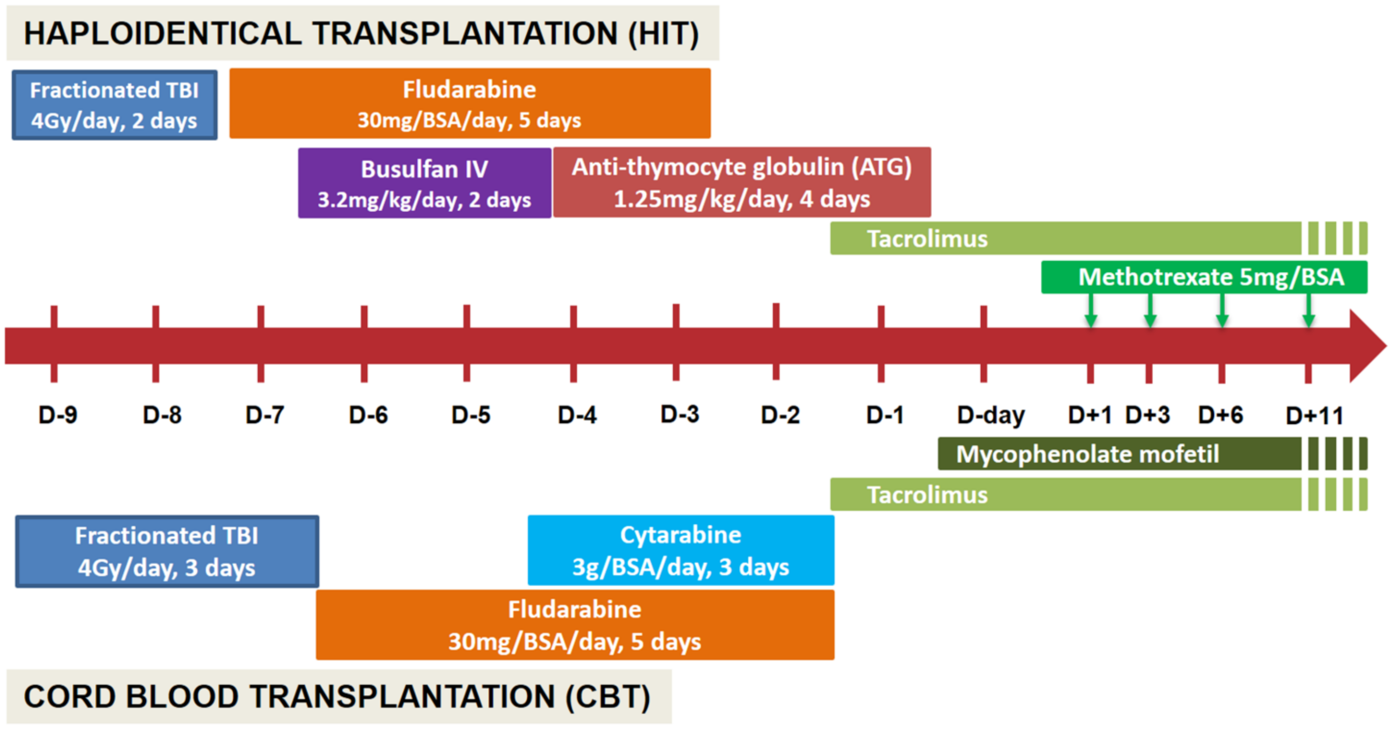

Figure S1. Conditioning scheme of haploidentical and cord blood transplantation

* TBI dose of 400 cGy for was omitted for 6 frail patients (5 in HIT group; 3 with age over 55 years old, 1 with bronchiolitis obliterans from SCT1, 1 with clinician's discretion; 1 in CBT group with age over 55 years old)

Abbreviations: BSA: body surface area; Gy: Gray; IV; intravenous; TBI: total body irradiation

Variables	HR	95% CI	p value
**Overall Survival**			
HIT versus dCBT	0.46	0.23—0.89	0.021
Age in decades	1.28	0.97—1.69	0.082
Female versus male	0.93	0.48—1.79	0.816
Poor cytogenetic risk	1.70	0.79—3.64	0.175
Autologous versus allogeneic SCT1	0.54	0.25—1.20	0.130
Relapse after ≥9 months from SCT1	0.55	0.27—1.10	0.090
HCT-CI High (≥3)	1.11	0.57—2.16	0.757
Remission at SCT2	0.48	0.22—1.05	0.066
Prior GVHD from SCT1*	1.08	0.49—2.39	0.844
**Disease-free Survival**			
HIT versus dCBT	0.47	0.24—0.92	0.027
Age in decades	1.23	0.94—1.62	0.135
Female versus male	0.83	0.43—1.59	0.575
Poor cytogenetic risk	1.57	0.73—3.38	0.245
Autologous versus allogeneic SCT1	0.49	0.22—1.07	0.072
Relapse after ≥9 months from SCT1	0.56	0.28—1.12	0.102
HCT-CI High (≥3)	1.02	0.53—1.97	0.943
Remission at SCT2	0.52	0.24—1.13	0.097
Prior GVHD from SCT1*	0.90	0.41—1.98	0.801
**Non-relapse mortality**			
HIT versus dCBT	0.34	0.14—0.84	0.019
Age in decades	1.57	1.15—2.16	0.005
Female versus male	1.11	0.46—2.68	0.814
Poor cytogenetic risk	1.67	0.64—4.40	0.298
Autologous versus allogeneic SCT1	0.57	0.21—1.55	0.271
Relapse after ≥9 months from SCT1	1.05	0.39—2.86	0.920
HCT-CI High (≥3)	1.29	0.54—3.07	0.562
Remission at SCT2	2.21	0.52—9.50	0.284
Prior GVHD from SCT1*	0.55	0.19—1.62	0.276
**Relapse**			
HIT versus dCBT	1.27	0.49—3.32	0.623
Age in decades	0.81	0.56—1.17	0.264
Female versus male	0.67	0.27—1.66	0.382
Poor cytogenetic risk	1.17	0.42—3.28	0.768
Autologous versus allogeneic SCT1	0.74	0.24—2.27	0.604
Relapse after ≥9 months from SCT1	0.46	0.18—1.18	0.106
HCT-CI High (≥3)	0.68	0.27—1.67	0.396
Remission at SCT2	0.25	0.10—0.63	0.003
Prior GVHD from SCT1*	1.54	0.55—4.31	0.416

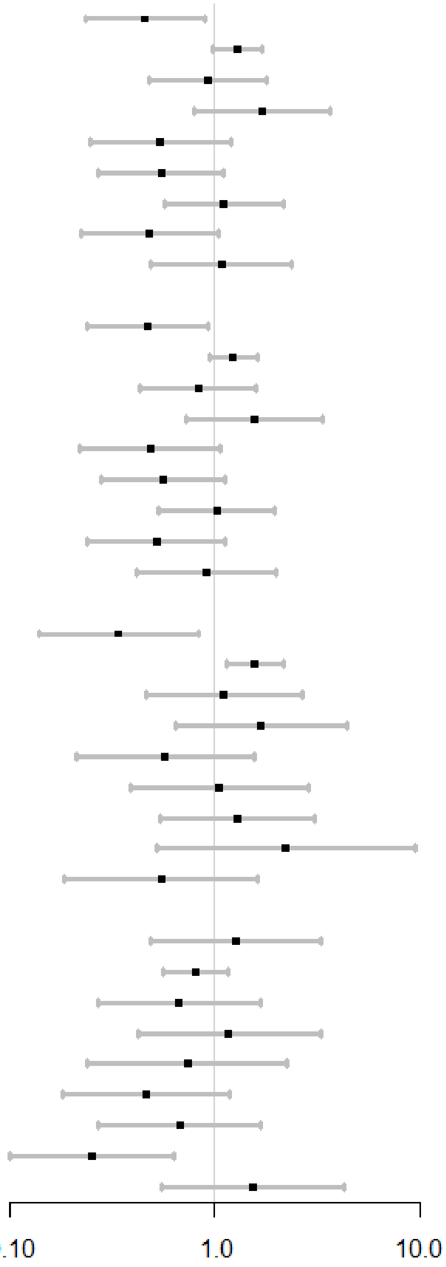

Figure S2. Univariate analysis for risk factors associated with SCT2 outcomes

Abbreviations: dCBT: double-cord blood transplantation; GVHD: graft-versus-host disease; HCT-CI: hemotopoietic cell transplantation-specific comorbidity index; HIT: haploidentical donor transplant; SCT1: first stem cell transplantation; SCT2: second stem cell transplantation. *History of either acute GVHD grade 2–4 or moderate to severe chronic GVHD

Variables	HR	95% CI	*p* value
**Overall Survival**			
HIT versus dCBT	0.31	0.13—0.70	0.005
Age in decades	1.24	0.88—1.75	0.218
Female versus male	1.16	0.52—2.59	0.725
Poor cytogenetic risk	2.35	0.97—5.70	0.058
Autologous versus allogeneic SCT1	0.52	0.19—1.40	0.197
Relapse after ≥9 months from SCT1	0.49	0.22—1.11	0.085
HCT-CI High (≥3)	1.22	0.54—2.72	0.633
WT1 PCR >250 copies/10^4 *ABL*	2.96	1.23—7.12	0.016
**Disease-free Survival**			
HIT versus dCBT	0.30	0.13—0.69	0.005
Age in decades	1.16	0.83—1.64	0.384
Female versus male	1.05	0.47—2.32	0.909
Poor cytogenetic risk	2.44	1.00—5.92	0.049
Autologous versus allogeneic SCT1	0.47	0.18—1.26	0.133
Relapse after ≥9 months from SCT1	0.50	0.22—1.15	0.104
HCT-CI High (≥3)	1.11	0.50—2.44	0.788
WT1 PCR >250 copies/10^4 *ABL*	3.47	1.44—8.38	0.006
**Non-relapse mortality**			
HIT versus dCBT	0.37	0.13—1.01	0.052
Age in decades	1.52	1.04—2.23	0.030
Female versus male	1.71	0.63—4.62	0.289
Poor cytogenetic risk	1.85	0.64—5.36	0.257
Autologous versus allogeneic SCT1	0.61	0.20—1.87	0.388
Relapse after ≥9 months from SCT1	0.96	0.33—2.76	0.943
HCT-CI High (≥3)	1.27	0.49—3.33	0.623
WT1 PCR >250 copies/10^4 *ABL*	1.01	0.28—3.61	0.985
**Relapse**			
HIT versus dCBT	0.72	0.23—2.29	0.579
Age in decades	0.74	0.44—1.25	0.260
Female versus male	0.42	0.11—1.51	0.182
Poor cytogenetic risk	1.82	0.52—6.37	0.351
Autologous versus allogeneic SCT1	0.60	0.13—2.65	0.497
Relapse after ≥9 months from SCT1	0.40	0.13—1.31	0.131
HCT-CI High (≥3)	0.69	0.22—2.18	0.526
WT1 PCR >250 copies/10^4 *ABL*	4.87	1.50—15.8	0.008

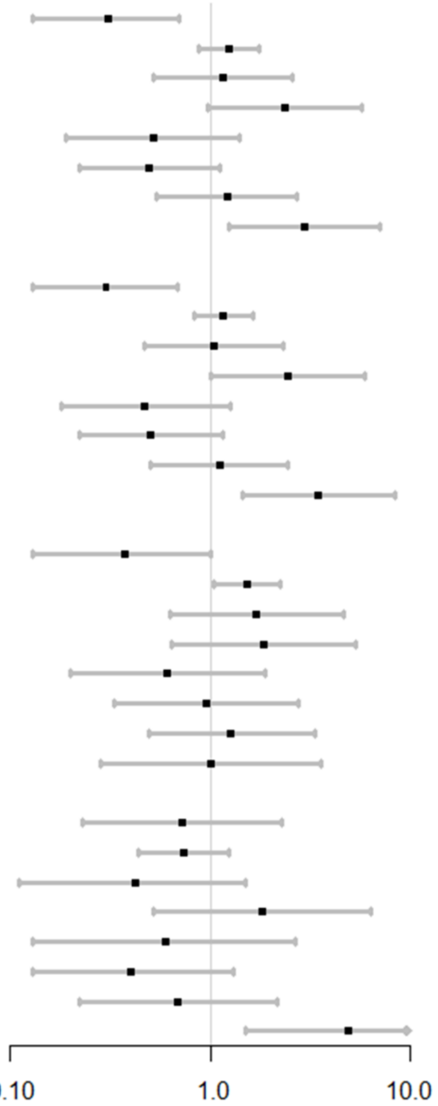

Figure S3. Univariate analysis for risk factors associated with SCT2 outcomes for patients in complete remission before SCT2 with available WT1-PCR ($N = 38$).

Abbreviations: ABL: Abelson gene; CI: confidence interval; dCBT: double-cord blood transplantation; HCT-CI: hemotopoietic cell transplantation-specific comorbidity index; HIT: haploidentical donor transplant; HR: hazard ratio; PCR: polymerase chain reaction; SCT1: first stem cell transplantation; SCT2: second stem cell transplantation; WT1: Wilms tumor 1 gene

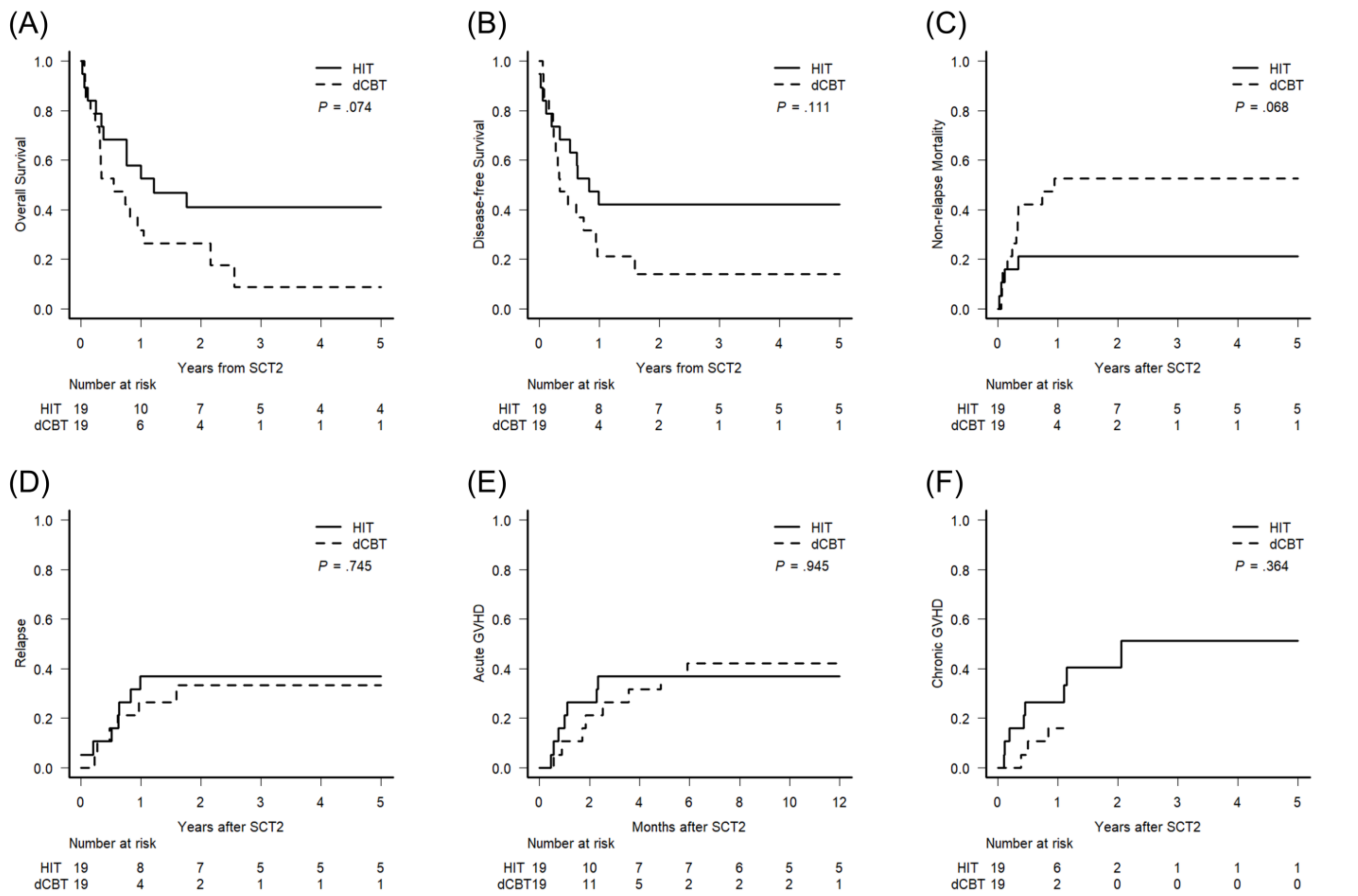

Figure S4. Transplant outcomes of subgroup of patients who received first transplant from allogeneic donor.

Abbreviations: dCBT: double-cord blood transplantation; GVHD: graft-versus-host disease; HIT: haploidentical donor transplant; SCT2: second stem cell transplantation

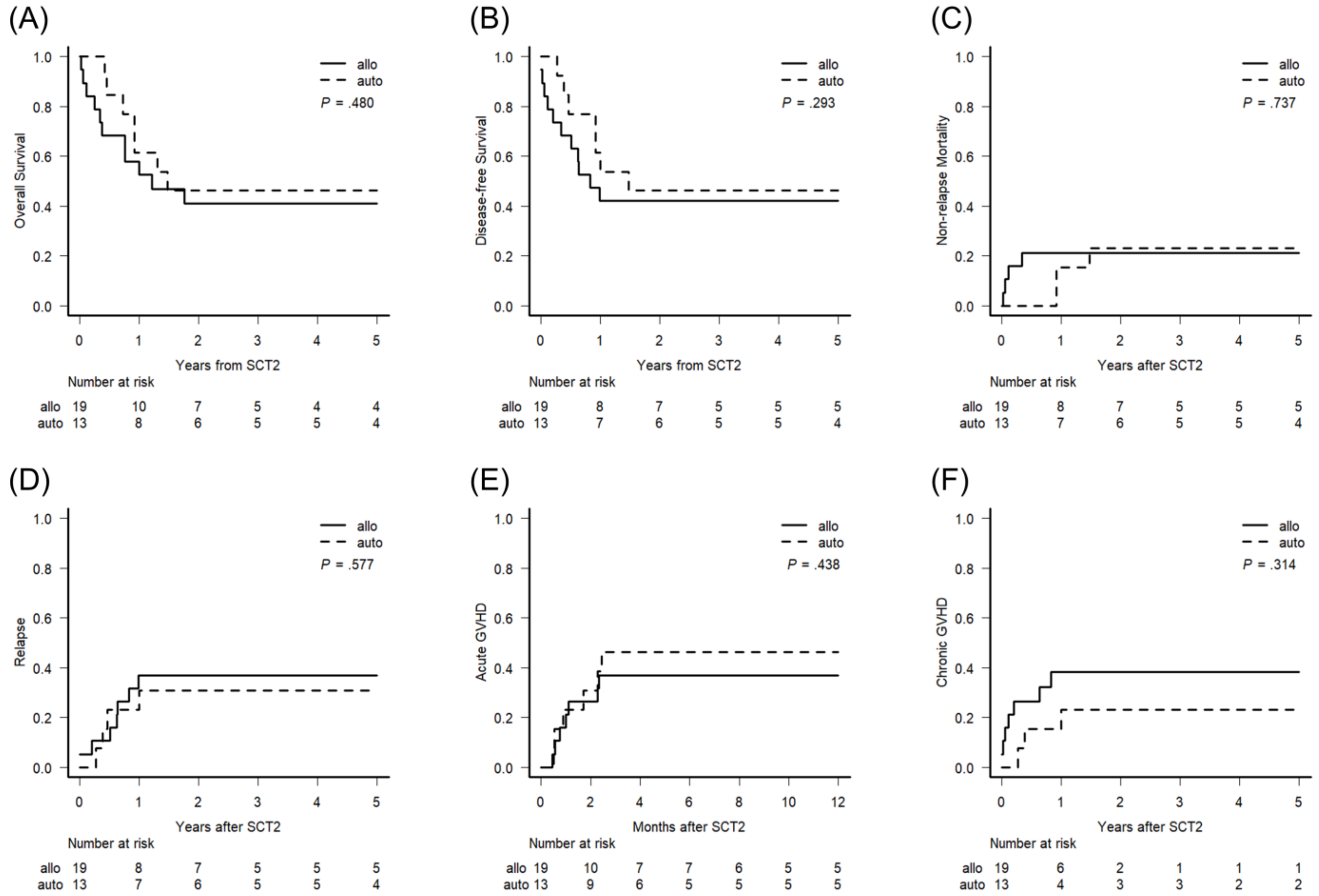

Figure S5. Transplant outcomes of subgroup of patients who received second stem cell transplant from haploidentical donor.

Abbreviations: allo: allogeneic stem cell transplantation; auto: autologous stem cell transplantation; GVHD: graft-versus-host disease; SCT2: second stem cell transplantation

Table S1. Complications after second transplantation

	**HIT** (*N* = 32)<br>*N* (%)	**dCBT** (*N* = 20)<br>*N* (%)	*p* value
CMV DNAemia (%)	24 (75)	15 (75)	1.000
CMV disease (%)	5 (15)	4 (20)	0.719
CMV treatment (%)	19 (59)	12 (60)	1.000
Hemorrhagic cystitis (%)	10 (31)	1 (5)	0.035
Thrombotic microangiopathy (%)	2 (6)	1 (5)	1.000
Sinusoidal obstruction syndrome (%)	2 (6)	0 (0)	0.517
Herpes zoster infection (%)	7 (22)	4 (20)	1.000

Abbreviations: dCBT: double cord blood transplantation; CMV: cytomegalovirus; HIT: haploidentical transplantation

Table S2. Causes of non-relapse mortality (NRM)

	**HIT** (*N* = 32)<br>*N* (%)	**dCBT** (*N* = 20)<br>*N* (%)	*p* value
Early NRM (<100 days from transplant)	3 (9)	5 (25)	0.235
Infection	2 (6)	4 (20)	0.189
SOS	1 (3)	0 (0)	1.000
Diffuse alveolar hemorrahge	0 (0)	1 (5)	1.000
Transplantation-related (%)			
Death in aplasia	1 (3)	2 (10)	0.551
Chronic GVHD	3 (9)	1 (5)	1.000
Hemorrhage	0 (0)	1 (5)	0.385
SOS	1 (3)	0 (0)	1.000
Infection	5 (16)	7 (35)	0.175
Lower respiratory infection	4 (13)	4 (20)	0.695
CNS infection	0 (0)	1 (5)	0.385
Bacterial sepsis	1 (3)	2 (10)	0.551
Other	0 (0)	1 (5)†	0.385

Abbreviations: CNS: Central nervous system; dCBT: double cord blood transplantation; GVHD: graft-versus-host disease; HIT: haploidentical transplantation; SOS: sinusoidal obstruction syndrome

† myelopathy of unidentified cause

Table S3. Comparison of outcomes after second haploidentical transplantation for relapsed AML

Reference	Study type	*N*	Disease type *N* (%)	Donor of SCT1 *N* (%)	Months from SCT1 to relapse median (range)	GVHD before SCT2	Salvage therapy *N* (%)	CR at SCT2 *N* (%)	Regimen intensity	Cell source	GVHD prophylaxis	Outcomes	GVHD incidence
Tischer et al.<br><br>BMT, 2014[1]	Retrospective, two centers<br><br>(Germany)	20	AML 15 (75)<br>ALL 4 (20)<br>AL, bi-phenotypic 1 (5)	MRD 6 (30)<br>MMRD 2 (10)<br>MUD 12 (60)	11 (1-54)	Not available	Cytoreduction 17 (85)<br>none 3 (15)	3 (15)	RIC = 20	PB = 6<br>BM = 14	PTCy<br>FK, MMF	1y-OS: 45%<br><br>1y-DFS: 33%<br><br>Relapse 7/20<br><br>1y-NRM: 36%	aGVHD: 12/20<br><br>cGVHD: 5/20
Srour et al. AJH. 2020[2]	Retrospective, single center<br><br>(MDACC)	29	AML 20 (70)<br>MDS 3 (10)<br>ALL 4 (14)<br>HD 2 (7)	MRD 7 (24)<br>MUD 10 (35)<br>Haplo 3 (10)<br>MMUD 2 (7)<br>CB 6 (21)<br>missing 1 (4)	9 (3-44)	Not available	Not available	7 (24)	MAC = 15<br>RIC = 14	PB = 9<br>BM = 20	PTCy<br>FK, MMF	3y-OS: 40%<br><br>3y-DFS: 31%<br><br>3y-Relapse: 30%<br><br>3y-NRM: 39%	aGVHD(100d): 45%<br><br>cGVHD(1 year): 7.2%
Kharfan-Dabaja et al.<br><br>BJH. 2021[3]	Retrospective, registry<br><br>(EBMT)	135	AML 135 (100)	Same donor 9 (7)<br>Different donor 125 (93)<br>missing 1 (4)	11 (1-89)	aGVHD Gr 2-4: 10%<br><br>cGVHD (any): 15%	Not available	61 (45)	MAC = 62<br>RIC = 72<br>missing = 1	PB = 29<br>BM = 106	PTCy otherwise not specified	2y-OS: 29%<br><br>2y-DFS: 29%<br><br>2y-Relapse: 48%<br><br>2y-NRM: 27%	aGVHD(180d, Gr 2-4):<br><br>27%<br><br>cGVHD (2 year): 22%
Current study	Retrospective, single center<br><br>(Seoul St. Mary's Hospital)	32	AML 32 (100)	Auto 13 (41)<br>MRD 11 (34)<br>MUD 7 (22)<br>CB 1 (3)	12 (3-137)	aGVHD Gr 2-4: 9%<br><br>cGVHD (moderate or severe): 9%	Intensive 30 (94)<br>Non-intensive 2 (6)	25 (78)	RTC = 32	PB = 32	ATG<br>FK, MTX	2y-OS: 43%<br><br>2y-DFS: 44%<br><br>2y-Relapse: 34%<br><br>2y-NRM: 22%	aGVHD(180d): 41%<br><br>cGVHD (2 year): 46%

Table S3. Comparison of outcomes after second haploidentical transplantation for relapsed AML (continued)

Reference	Study type	*N*	Factors associated with poor outcomes			
			OS	DFS	Relapse	NRM
Tischer et al.<br><br>BMT, 2014[1]	Retrospective, two centers<br><br>(Germany)	20	Duration of remission after SCT1 (≤ 6 months)	-	-	-
Srour et al. AJH. 2020[2]	Retrospective, single center<br><br>(MDACC)	29	HCT-CI ≥ 3<br><br>DSA	HCT-CI ≥ 3<br><br>DSA	Duration of remission after SCT1 (≤ 6 months)	HCT-CI ≥ 3<br><br>DSA
Kharfan-Dabaja et al.<br><br>BJH. 2021[3]	Retrospective, registry<br><br>(EBMT)	135	Active disease (vs CR)<br><br>Older age<br><br>Duration of remission after SCT1 (≤ 13.2 months)	Active disease (vs CR)<br><br>Duration of remission after SCT1 (≤ 13.2 months)	Active disease (vs CR)<br><br>Duration of remission after SCT1 (≤ 13.2 months)<br><br>Patient CMV seropositivity	Duration of remission after SCT1 (≤ 13.2 months)
Current study	Retrospective, single center<br><br>(Seoul St. Mary's Hospital)	32	Older age	-	Duration of remission after SCT1 (≤ 9 months)<br><br>Active disease (vs CR)	Older age

Abbreviations: AJH: American Journal of Hematology; aGVHD: acute graft-versus-host disease; AL: acute leukemia; ALL: acute lymphoblastic leukemia; AML: acute myeloid leukemia; ATG: antithymocyte globulin rabbit (Thymoglobuline®); BJH: British Journal of Haematology; BMT: Bone Marrow Transplantation; cGVHD: chronic graft-versus-host disease; CMV: cytomegalovirus; CR; complete remission; DFS: disease-free survival; DSA, donor-specific anti-HLA antibodies; EBMT: The European Society for Blood and Marrow Transplantation; FK: tacrolimus; Gr: grade; HCT-CI, hematopoietic cell transplant-comorbidity index; HD: Hodgkin disease; HIT: haploidentical transplantation; MAC: myeloablative conditioning; MDACC: MD Anderson Cancer Center; MDS: Myelodysplastic syndrome; MMF: mycophenolate mofetil; MTX: methotrexate; NRM: non-relapse mortality; OS: overall survival; PTCy: post-transplant cyclophosphamide; RIC: reduced intensity conditioning; RTC: reduced toxicity conditioning; SCT1: first stem cell transplantation; SCT2: second stem cell transplantation

1. Tischer J, Engel N, Fritsch S, et al. Second haematopoietic SCT using HLA-haploidentical donors in patients with relapse of acute leukaemia after a first allogeneic transplantation. *Bone Marrow Transplant.* 2014;49(7):895-901.
2. Srour SA, Kongtim P, Rondon G, et al. Haploidentical transplants for patients with relapse after the first allograft. *Am J Hematol.* 2020.
3. Kharfan-Dabaja MA, Labopin M, Brissot E, et al. Second allogeneic haematopoietic cell transplantation using HLA-matched unrelated versus T-cell replete haploidentical donor and survival in relapsed acute myeloid leukaemia. *Br J Haematol.* 2021;193(3):592-601.

Table S4. Characteristics of patients who received first transplant from allogeneic donor

**Characteristics**		**HIT (*N* = 19), *N* (%)**	**dCBT (*N* = 19), *N* (%)**	***p* value**
AML MRC		3 (16)	4 (21)	1.000
Age at SCT2, median (range)		47 (24-67)	47 (25-63)	0.942
Male		10 (53)	12 (63)	0.743
Cytogenetics[a]	Favorable	0 (0)	3 (16)	0.038
	Intermediate	17 (89)	10 (53)	
	Adverse	2 (11)	6 (32)	
FLT3-ITD	Positive	3 (16)	3 (15)	0.511
	Negative	10 (53)	14 (70)	
	Not available	6 (32)	3 (15)	
Donor at SCT1	Matched-related	11 (58)	4 (21)	<0.001
	Matched-unrelated	7 (37)	2 (11)	
	Haploidentical	0 (0)	13 (68)	
	Cord blood	1 (5)	0 (0)	
SCT1 Intensity	MAC	12 (63)	4 (21)	0.010
	RTC[b]	3 (16)	12 (63)	
	RIC	4 (21)	3 (16)	
GVHD post-SCT1	aGVHD grade ≥ 2	3 (16)	4 (21)	1.000
	cGVHD ≥ moderate	3 (16)	6 (32)	0.447
Remission duration after SCT1 ≥ 9 months		14 (74)	13 (68)	1.000
Salvage therapy	Intensive	17 (89)	17 (89)	1.000
	Non-intensive	2 (11)	2 (11)	
	With DLI	2 (11)	0 (0)	0.486
Complete remission before SCT2		15 (79)	16 (84)	1.000
WT1 PCR (/10^4 ABL) before SCT2				1.000
CR, ≥250 (MRD-positive)		3 (16)	3 (16)	
CR, <250 (MRD-negative)		11 (58)	12 (63)	
CR, but data unavailable		1 (5)	1 (5)	
Non-CR		4 (21)	3 (16)	
HCT-CI	0	0 (0)	5 (26)	0.007
	1-2	9 (47)	2 (11)	
	≥3	10 (53)	12 (63)	
Donor sex	Match	8 (42)	3 (16)	
	Partial mismatch	-	11 (58)	
	Full mismatch	11 (58)	5 (26)	
Donor HLA match (of eight loci, the lower value in dCBT)	4	8 (42)	3 (16)	<0.001
	5	7 (37)	7 (37)	
	6	4 (21)	6 (32)	
	≥7	0 (0)	3 (16)	
Total nucleated cells, median (range), 10^8/kg		20.1 (9.2-40.3)	0.39 (0.18-0.71)	<0.001
CD34, median (range), 10^6/kg		8.86 (4.37-28.96)	0.07 (0.03-0.32)	<0.001
Time between SCT1 and SCT2, median months (range)		17.5 (6.6-127.3)	15.4 (6.6-82.0)	0.280
Time between relapse and SCT2, median months (range)		3.7 (2.8-7.9)	3.7 (2.8-7.1)	0.977

Abbreviations: AML: acute myeloid leukemia; CR: complete remission; dCBT: double-cord blood transplantation; DLI: donor lymphocyte infusion; FLT3-ITD: fms-related tyrosine kinase 3-internal tandem duplication; GVHD: graft-versus-host disease; HCT-CI: hemotopoietic cell transplantation-specific comorbidity index; HIT: haploidentical donor transplant; HLA: human leukocyte antigen; MAC: myeloablative conditioning; MRC: myelodysplasia-related changes; PCR: polymerase chain reaction; RIC: reduced-intensity conditioning; RTC: reduced-toxicity conditioning; SCT1: first stem cell transplantation; SCT2: second stem cell transplantation; TNC: total nucleated cells. [a]Cytogenetic classification was based on the International Working Group (IWG) response criteria for acute myeloid leukemia, published in 2003. [b]Reduced toxicity conditioning refers to fractionated TBI (8.0 Gy), fludarabine (150 mg/m2), and intravenous busulfan (6.4 mg/kg).
